# Bridge Plating Versus Hybrid External Fixation in the Management of Proximal Tibia Metaphyseal Fractures

**DOI:** 10.7759/cureus.36331

**Published:** 2023-03-18

**Authors:** Ramavtar Saini, Anshu Sharma, Shehbaz S Sidhu, Kuldeep S Rathore

**Affiliations:** 1 Orthopaedics, Geetanjali Medical College and Hospital, Udaipur, IND

**Keywords:** knee society score, hybrid external fixator, bridge plating, metaphyseal fracture, proximal tibia fracture

## Abstract

Background: Due to the surge in high-speed road traffic accidents during the past few years, extra-articular proximal tibia fractures have also risen in number. For the treatment of such fractures, various options are available like conservative treatment with casting, surgical treatment with plate osteosynthesis, or a hybrid external fixator. Exposure of the bone surface and extensive soft tissue dissection is needed in bridge plating, posing a risk of bleeding, infection, and soft-tissue healing issues, and the blood supply of the fractured area is also hampered as the periosteum is also destroyed. To avoid these complications, a hybrid external fixator can be used but it has its own risks of malunion, non-union, and pin-tract infections; another drawback is patient compliance. In this study, we compare the clinical and functional outcomes of two treatment modalities, i.e., bridge plating and hybrid external fixator, in the treatment of proximal tibia metaphyseal fractures.

Materials and methods: This prospective randomized study was conducted from February 2021 to June 2022 on 46 adult patients diagnosed with proximal tibia metaphyseal fracture and willing to participate. An odd number of patients were treated with a bridge plate and even with a hybrid external fixator.

Results: A total of 46 patients were included in the study, sustaining proximal tibia metaphyseal fracture, out of which 23 patients underwent hybrid external fixation with an outcome of 69.43 ± 8.11 according to the Knee Society Score (KSS) and 23 for bridge plating which showed better results as their score at final follow-up was 75.00 ± 8.22.

Conclusion: According to our study, we found that bridge plating is a better treatment modality than the hybrid external fixator as the former showed better postoperative knee range of motion and functional outcome and led to a smaller number of complications. But the clinical outcome would also be influenced by the type of fracture, degree of comminution, type of injury, i.e., open or closed, and quality of bone.

## Introduction

Extra-articular proximal tibia fractures are common and account for about 5-11% of all tibial fractures [1]. Due to its superficial position and the rise of high-speed motor vehicle accidents over the past few years frequency of tibia fractures is also increasing [2,3]. High-energy fractures and low-energy fractures are the two main categories under which these fractures can be divided. Metaphyseal fractures of the proximal tibia usually occur secondary to direct bending forces applied to the region of the upper leg. Treatment options for metaphyseal tibial fractures include intramedullary interlocking nailing, plate osteosynthesis, external fixator application, and conservative therapy. Both benefits and drawbacks are unique to each treatment modality.

Fixation of extra-articular proximal tibia fractures with bridge plating has demonstrated excellent results as it can restore the anatomic structure of the bone [4]. But plating requires exposure of the bone surface and extensive soft tissue dissection, thus carrying the risks of bleeding, infection, soft-tissue healing problems, and hardware-related complications as well as it destroys the periosteum which is very important for blood supply to the fractured area [5]. Although problems related to soft-tissue dissection and healing can be largely sorted out with a hybrid external fixator (HEF), it bears the risks of malunion, non-union, and pin-tract infections. Another big limit to external fixators is patient compliance in accommodating the device as well as daily care of the pin sites.

This study was undertaken to compare the postoperative complications, and clinical and functional outcomes of proximal tibia metaphyseal fractures treated either with bridge plating or hybrid external fixation. The Knee Society Score (KSS) was used, which includes the parameters of pain, range of motion, stability, alignment, and extension lag for the assessment of functional outcomes [6].

## Materials and methods

This prospective randomized study was conducted at a tertiary care medical teaching institute in southern Rajasthan, India. Ethical committee clearance was obtained (Institutional Ethics Committee, Geetanjali Medical College and Hospital, Udaipur, Rajasthan: GU/HREC/EC/2021/1930). All patients who presented to the orthopedic outpatient department or casualty with proximal tibia metaphyseal fractures (closed or open up to Gustilo-Anderson grade III-B), over 18 years of age, and willing to give consent for participation in the study were included. Proximal tibia fractures with a neurovascular deficit of the injured limb (Gustilo-Anderson grade III-C), pathological fractures, or any associated bony injury on the same limb were excluded [7].

On presentation, the patients were screened for other system injury since they are closely associated with high-velocity trauma. Any associated neurovascular deficit was ruled out at this stage and taken note of the same. Gentle manipulation was done to bring gross reduction of the fracture segment and an above-knee support slab was applied. True anteroposterior and lateral radiographs were taken. On admission, detailed history of injury including the mode and severity of the injury was taken. Informed and written consent in their known language was taken from all the patients willing to participate in the study as per the guidelines of the institutional ethical committee before the initiation of data collection. All the fractures were classified using the Orthopaedic Trauma Association (OTA) classification [8].

According to the inclusion and exclusion criteria, 46 patients were enrolled in this study and were randomly divided into two groups based on their sequence of presentation with single blinding, groups A and B with 23 patients in each group. Group A patients underwent internal fixation with bridge plating. All the surgeries were performed using a minimally invasive percutaneous plate osteosynthesis (MIPPO) technique with a 3.5 mm locking compression plate (LCP) of the proximal tibia by an anterolateral approach. In group B, patients underwent fixation with HEF, beaded olive wires were inserted through safe zones of the proximal tibia and connected to a semi-circular frame, and tensioned using a tensioner. Two or three Schanz screws were inserted in the tibial diaphysis and connected with a tubular rod to each other as well as the semi-circular ring to complete the construct.

In the postoperative period, static quadriceps and ankle pump exercise were started on the same day or the next day of surgery. Knee range-of-motion exercises both active and passive were started as early as possible once the pain subsides and the patient feels comfortable, usually within two or three days after surgery. In the plating group, the surgical wound was inspected and dressed on the third postoperative day while in the HEF group, daily pin-tract dressing was done. Patients were discharged on the fifth to seventh postoperative day and antibiotics and analgesics were given as per requirement. On the 12th-14th postoperative day, the sutures were removed. Regular postoperative follow-up was done in the second week, sixth week, third month, and sixth month. At every follow-up visit, radiographs were obtained and the patients were clinically assessed for pain or any other complaints, and the functional outcome was evaluated according to the KSS [6].

The Student’s t-test or χ^2^ test was used to evaluate the differences between the groups. A p-value <0.005 was used as the cutoff for statistically significant differences.

## Results

In our study, there were 21 males (91.30%) and two females (8.70%) in the HEF group with the mean age being 40.48 ± 18.63 years, while in the bridge plating group there were 20 males (86.96%) and three females (13.04%) with the mean age being 46.91 ± 17.69 years. Both groups had no significant difference in the demographic profile (p > 0.005) (Table 1).

**Table 1:** Demographic variables of cases. The demographic variables of cases like age, gender, and side involvement in both the groups were comparable and no statistically significant difference was observed.

Variable	Bridge plating group	Hybrid external fixator group	p-Value
Age (in years)	46.91 ± 17.69	40.48 ± 18.63	>0.005
Sex: Male/Female	20/3	21/2	>0.005
Side involvement: Right/Left	15/8	14/9	>0.005

The bridge plating group consisted of 21 cases (91.3%) who had injuries resulting from a road traffic accident (RTA) while two cases (8.7%) had been hit by an animal. The HEF group also had 21 cases (91.3%) who were injured as a result of an RTA, but the remaining two cases came following a fall of a heavy object on the leg and a fall from height, respectively.

According to the OTA classification, in the ridge plating group, 15 patients had 41A2 and eight had 41A3 while in the HEF group, 12 patients had 41A2 and 11 had 41A3. In the bridge plating group 20 patients (86.96%) had closed injuries and three patients (13.04%) had open fractures, whereas in the HEF group, 19 patients (82.60%) had closed injuries and four patients (17.40%) had open fractures. Both groups had no significant difference in terms of the mechanism of injury and fracture pattern (p > 0.005) (Table 2).

**Table 2:** Mechanism of injury and fracture-related variables. Mechanism of injury and fracture-related variables in both the groups were comparable and no statistically significant difference was observed.

Variable	Bridge plating group	Hybrid external fixator group	p-Value
Mechanism of injury			>0.005
Road traffic accidents	21 (91.30%)	21 (91.30%)	
Hit by animal	O2	00	
Fall of heavy object	00	01	
Fall from height	00	01	
Classification (Orthopaedic Trauma Association)			>0.005
41A2	15 (65.21%)	12 (52.17%)	
41A3	08 (34.79%)	11 (47.83%)	
Type of fracture			>0.005
Open fracture	03 (13.04%)	04 (17.40%)	
Close fracture	20 (86.96%)	19 (82.60%)	

The mean surgery time (minutes) in the HEF group was 60 ± 9.53 minutes and in the bridge plating group was 71.09 ± 10.76 minutes. There was a significant difference in mean surgery time between the two groups with a p-value of 0.001.

The mean partial weight bearing (PWB) time in the HEF group was 5.13 ± 3.4 weeks and in the bridge plating group was 7.74 ± 1.94 weeks. The mean full weight bearing (FWB) time in the HEF group was 9.13 ± 3.4 weeks and in the bridge plating group was 12.43 ± 2.76 weeks. There was a significant difference in mean PWB and FWB time between the two groups. Four patients in the HEF group and two cases in the bridge plating group had extension loss (Table 3).

**Table 3:** Intraoperative and postoperative variables.

Variable	Bridge plating group	Hybrid external fixator group	p-Value
Mean surgery time (minutes)	71.09 ± 10.76	60 ± 9.53	<0.005
Mean hospital stay (days)	05.17 ± 1.47	05.48 ± 1.65	>0.005
Mean mobilization time (weeks)			
Partial weight bearing	07.74 ± 1.94	05.13 ± 3.4	<0.005
Full weight bearing	12.43 ± 2.76	09.13 ± 3.4	<0.005
Extension lag present in	2 cases (8.70%)	4 cases (17.39%)	>0.005

On regular follow-up, we observed that in the HEF group, one case (4.35%) had delayed union, five cases (21.74%) had a pin-tract infection, and 17 cases (73.91%) had no complications. In the bridge plating group, one case (4.35%) had screw back out, two cases (8.7%) had superficial infection, one case (4.35%) had a wound gaping, and 19 cases (82.61%) had no complication (Table 4).

**Table 4:** Complications distribution between two groups.

Complications	Hybrid external fixator group		Bridge plating group	
Delayed union	01	4.35%	00	0.00%
Pin-tract infection	05	21.74%	00	0.00%
Screw backout	00	0.00%	01	4.35%
Superficial infection	00	0.00%	02	8.70%
Wound gaping	00	0.00%	01	4.35%
None	17	73.91%	19	82.61%

The mean KSS in the HEF group was 69.43 ± 8.11 and in the bridge plating group was 75.00 ± 8.22. In the HEF group, 13.04% had excellent outcomes, 30.43% had good, 43.48% had fair, and 13.04% had poor outcome. In the bridge plating group, 30.43% had excellent outcomes, 43.48% had good, 21.74% had fair, and 4.35% had poor outcome.

**Figure 1:**
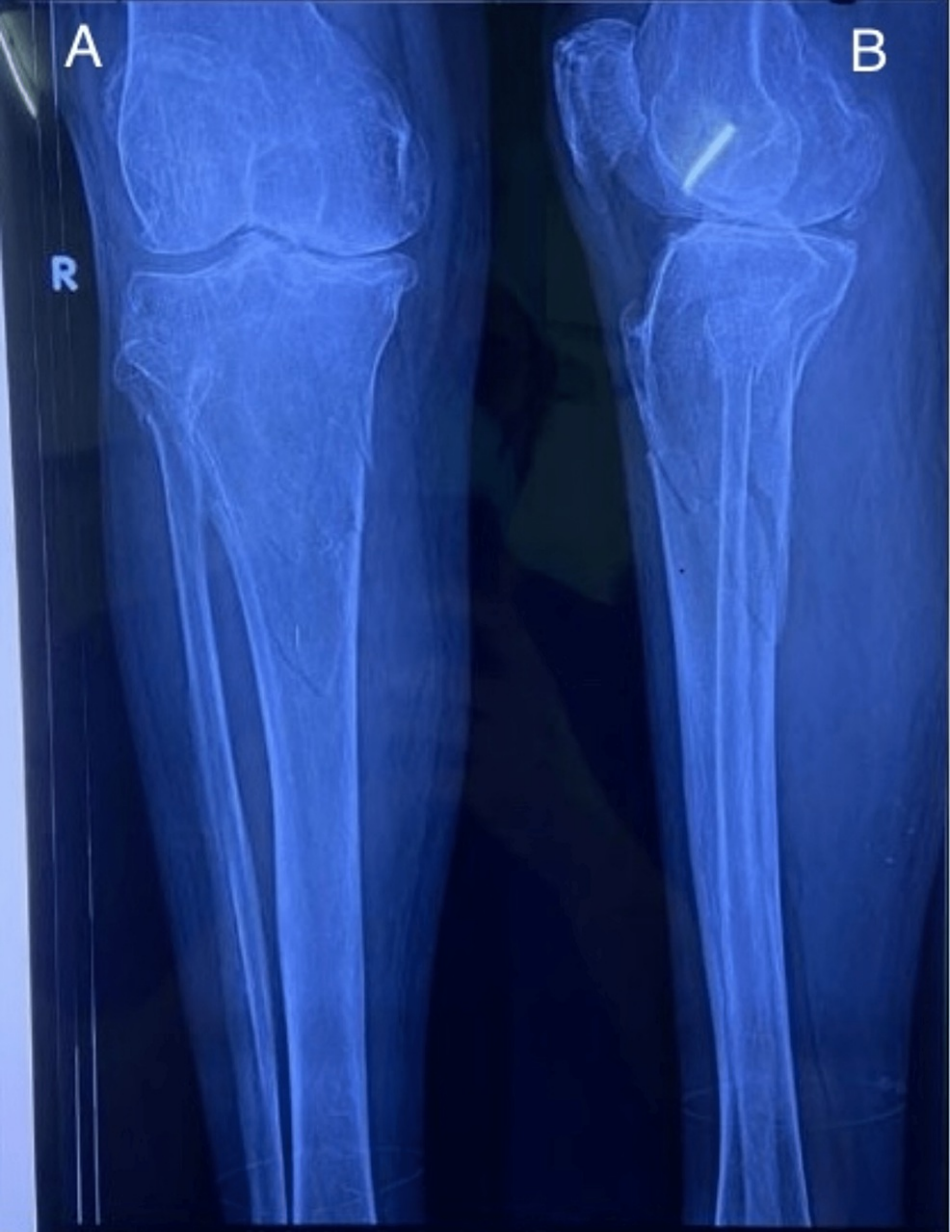
Preoperative radiograph showing proximal tibia metaphyseal fracture. Preoperative radiograph showing metaphyseal fracture of proximal tibia. Figure A is an anteroposterior (AP) and Figure B is a lateral view.

**Figure 2:**
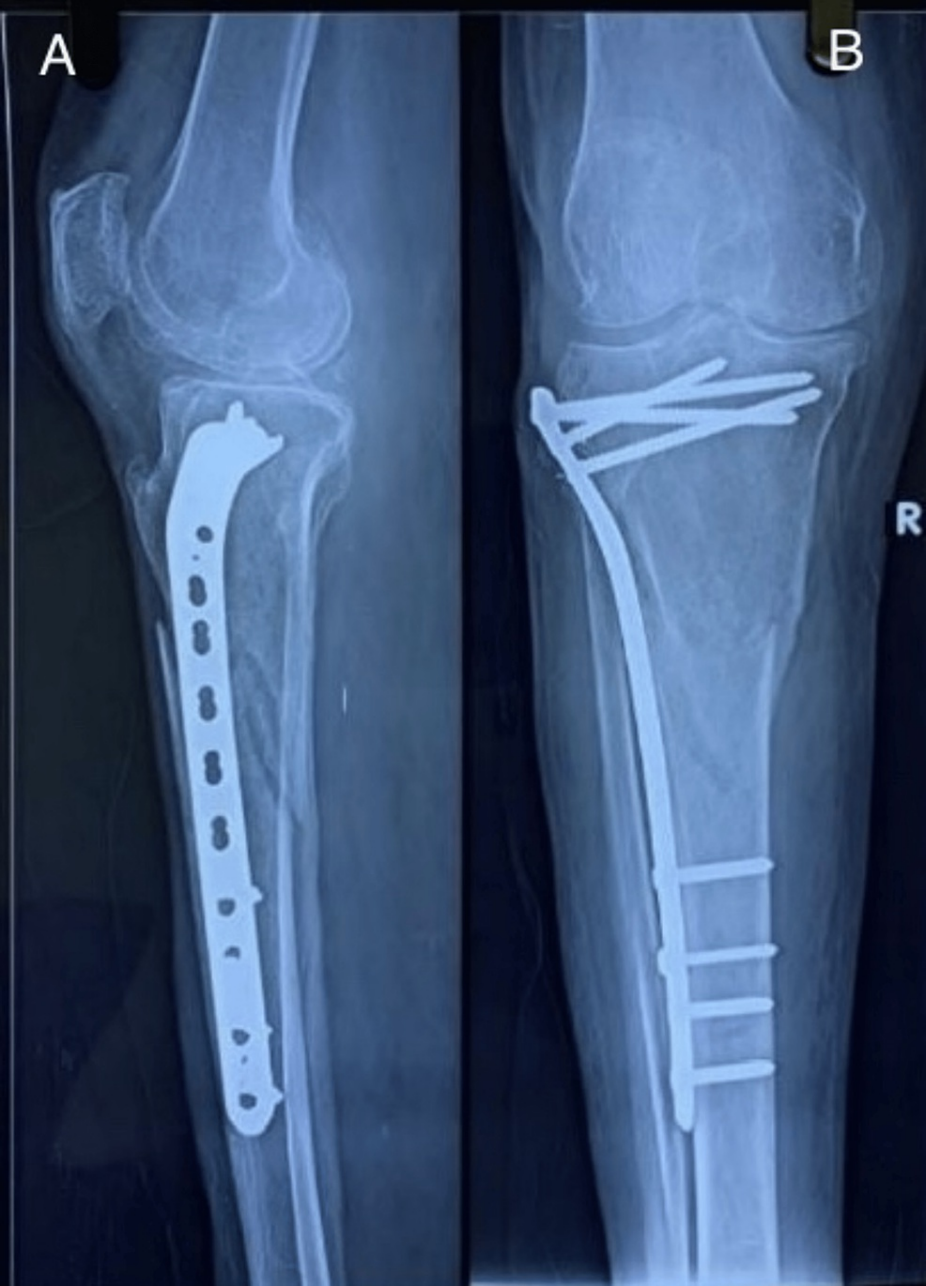
Postoperative radiograph. Postoperative radiograph showing fixation of fracture with locking plate. Figure A is an anteroposterior (AP) and Figure B is a lateral view.

**Figure 3:**
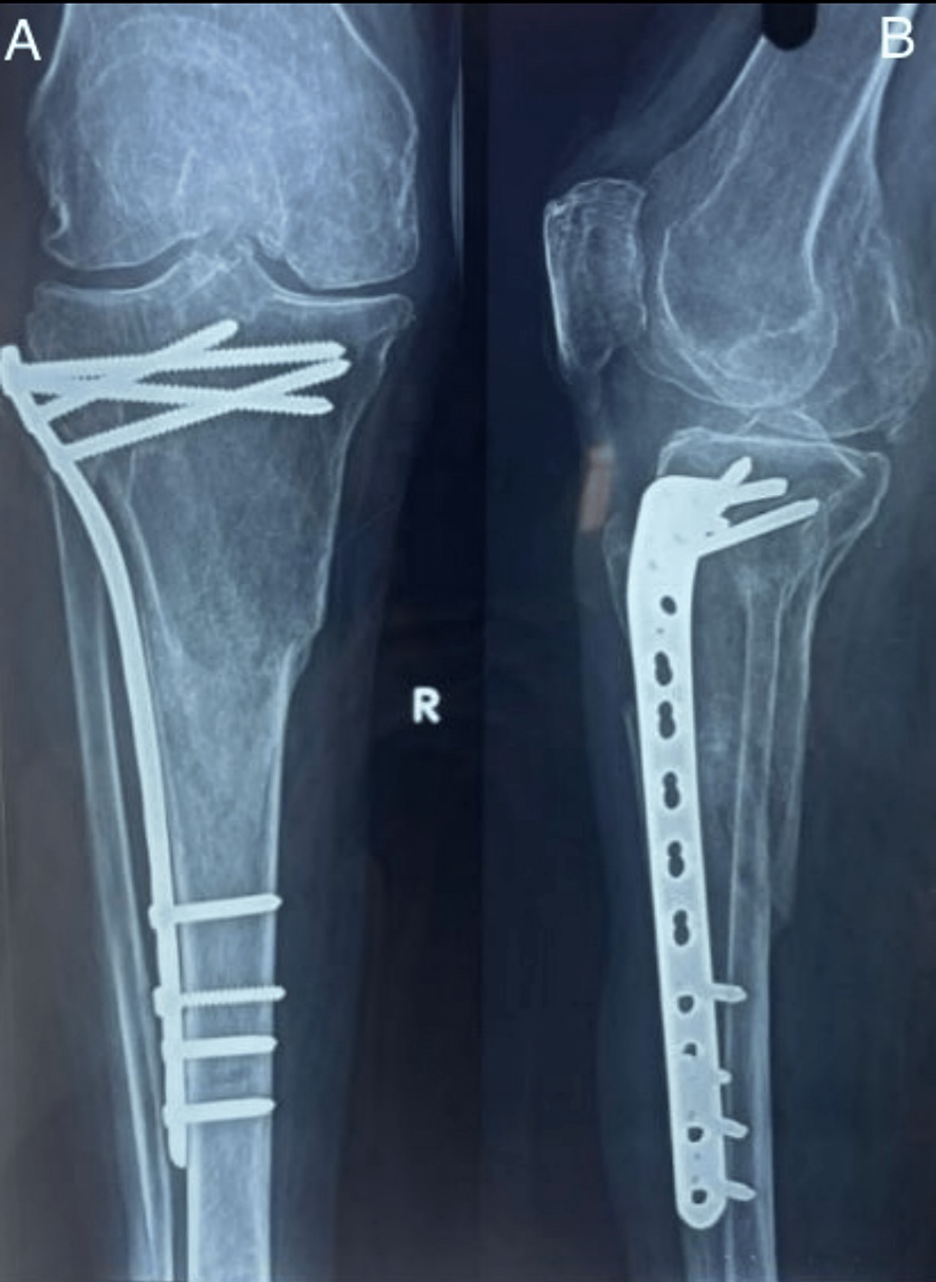
Sixth-month postoperative follow-up radiograph. Final follow-up radiograph showing fracture union. Figure A is an anteroposterior (AP) and Figure B is a lateral view.

**Figure 4:**
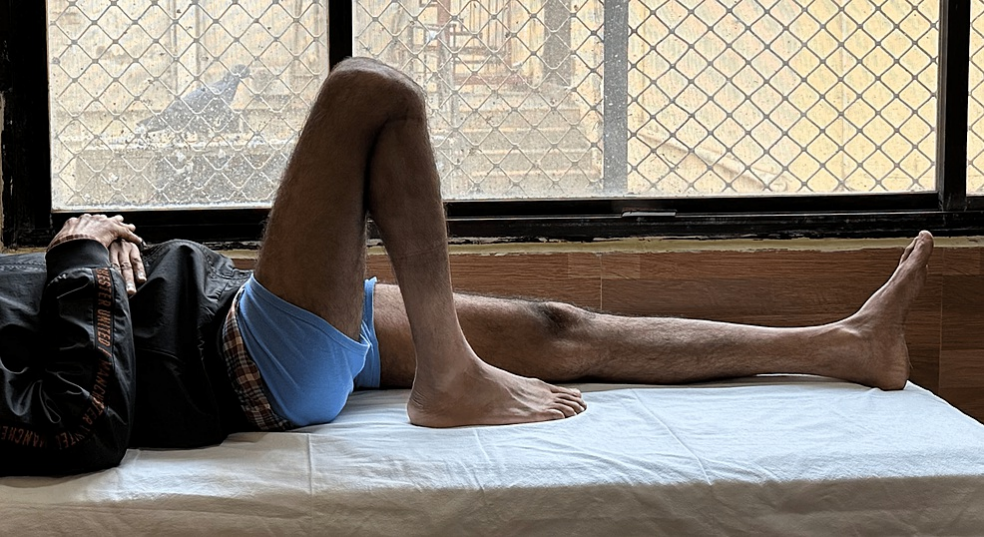
Clinical photograph showing flexion movement in the knee joint. Clinical photograph at final sixth-month postoperative follow-up showing good flexion range of movement in the knee joint.

**Figure 5:**
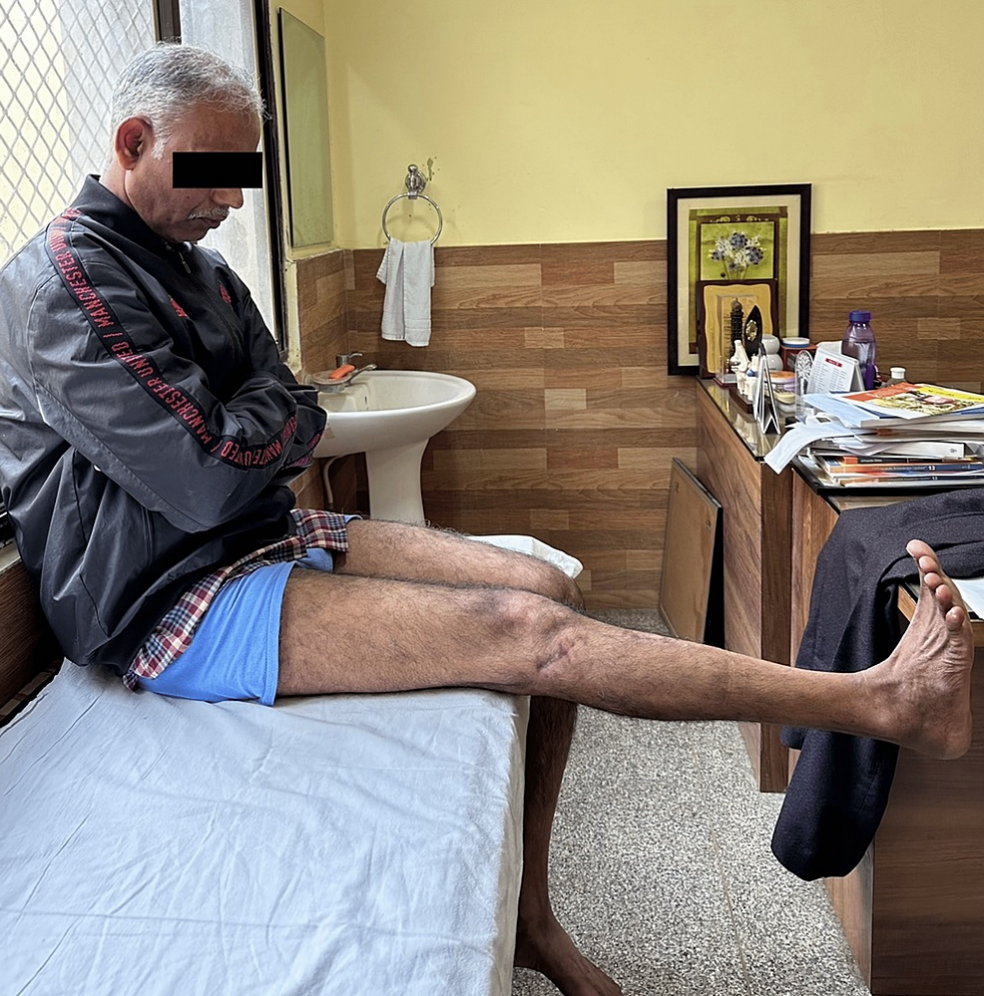
Clinical photograph showing active extension movement in the knee joint. Clinical photograph at the final sixth-month postoperative follow-up showing good active extension range of movement in the knee joint.

Following are the radiographic images of one of our study cases managed by bridge plating (Figures 1-3) and clinical images at the final follow-up (Figures 4, 5).

**Figure 6:**
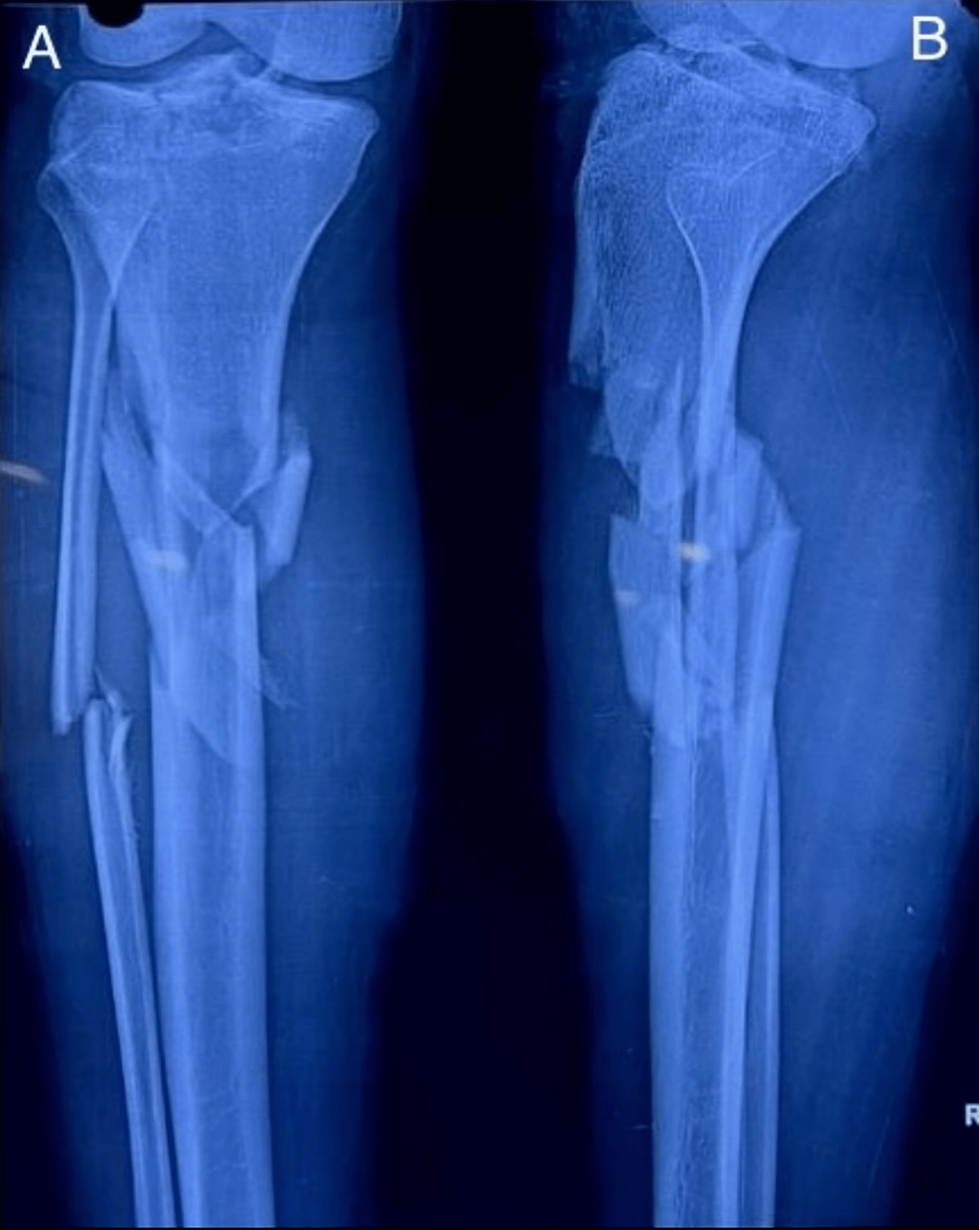
Preoperative radiograph. Preoperative radiograph showing metaphyseal comminuted fracture of proximal tibia. Figure A is an anteroposterior (AP) and Figure B is a lateral view.

**Figure 7:**
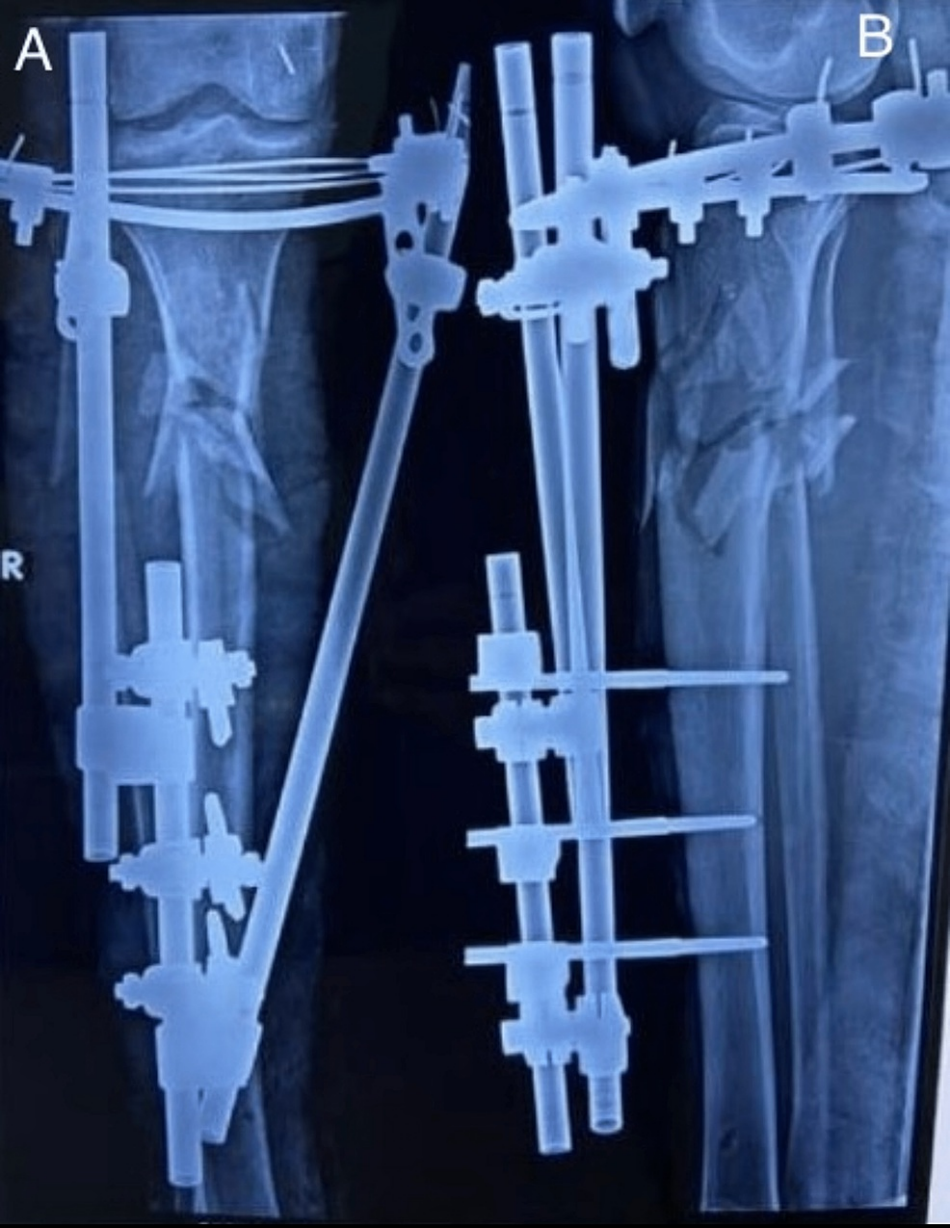
Postoperative radiograph. Postoperative radiograph showing fixation of fracture with hybrid external fixator. Figure A is an anteroposterior (AP) and Figure B is a lateral view.

**Figure 8:**
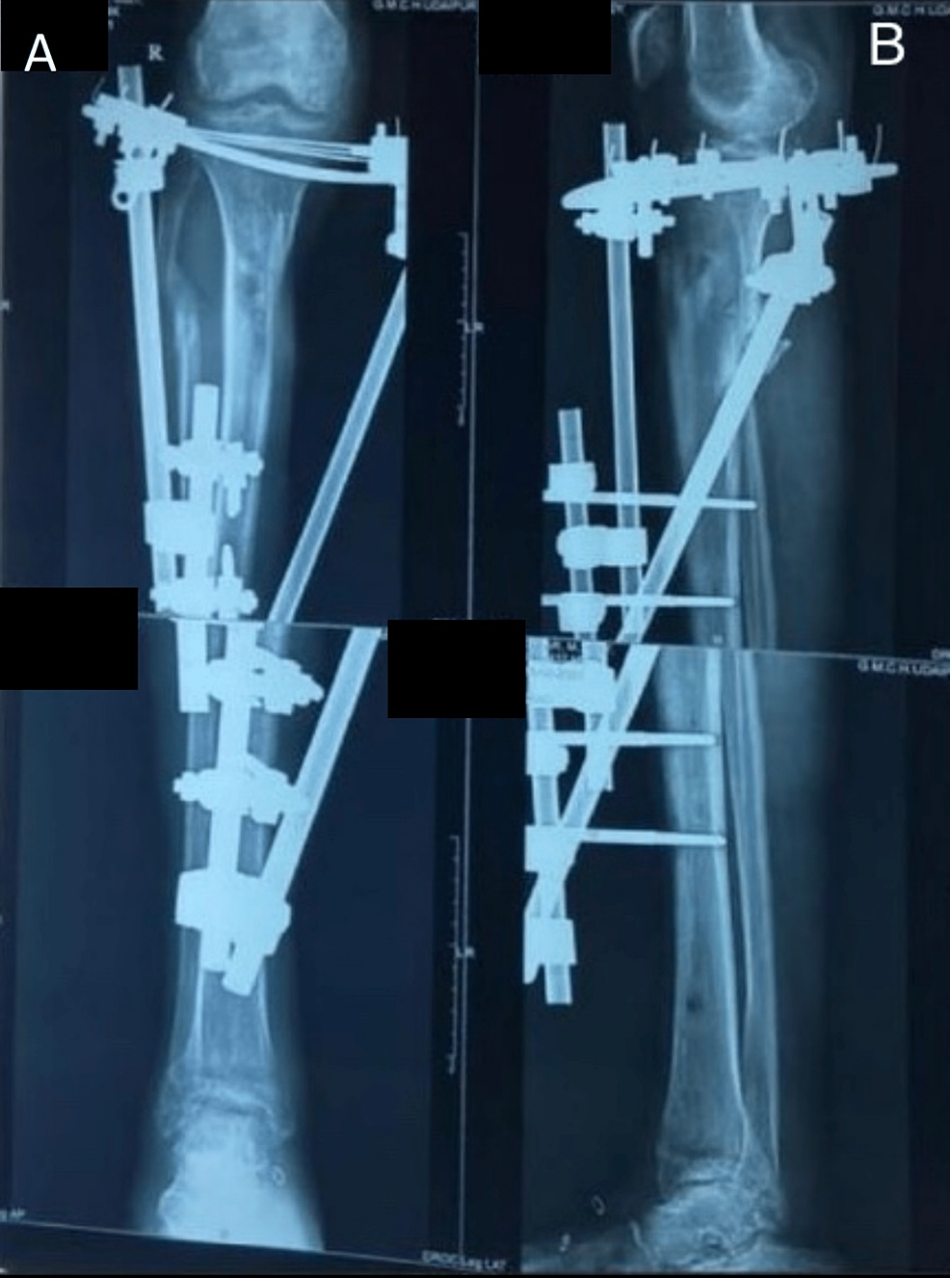
Sixth-month postoperative follow-up radiograph. Final follow-up radiograph showing fracture union. Figure A is an anteroposterior (AP) and Figure B is a lateral view.

**Figure 9:**
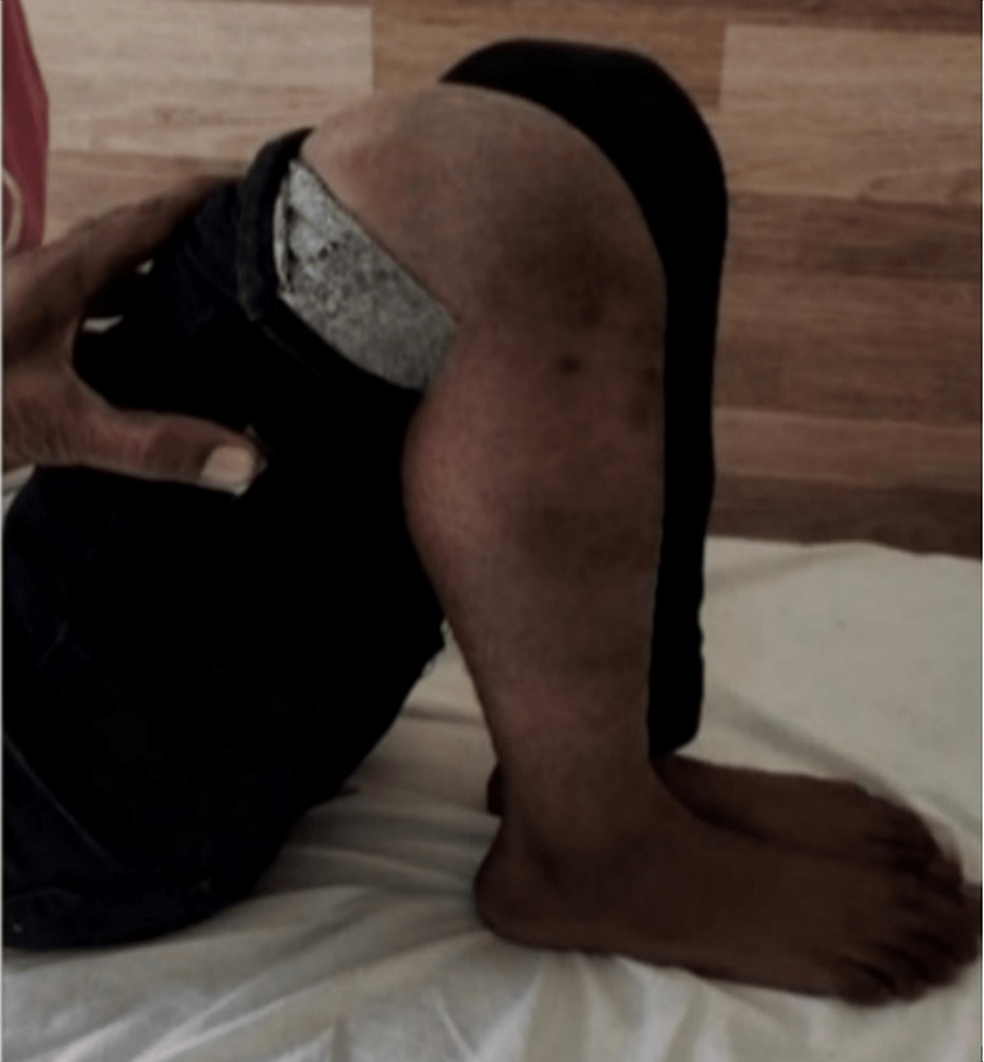
Clinical photograph showing flexion movement in the knee joint. Clinical photograph at the final sixth-month postoperative follow-up showing good flexion range of movement in the knee joint.

**Figure 10:**
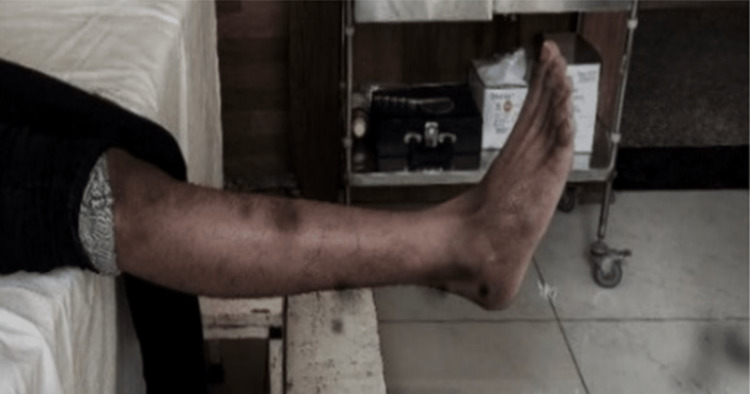
Clinical photograph showing extension movement in the knee joint. Clinical photograph at the final sixth-month postoperative follow-up showing good extension range of movement in the knee joint.

Following are the radiographic images of one of our study cases managed by HEF (Figures 6-8) and clinical images at the final follow-up (Figures 9, 10).

## Discussion

Proximal tibia fractures present a spectrum of soft-tissue and bony injuries that can produce permanent disability. There are many causes that lead to this fracture, but high-energy trauma remains the most common. The prevalence of these fractures is rising due to the increase in motor vehicles, so it is crucial to treat any knee injury properly because delaying care will result in serious morbidity and negatively impact the patient's quality of life [1]. Unsatisfactory, to be more precise, disappointing results have been produced with nonoperative treatment because of the major limitations of inadequate reduction and ineffective limb alignment [9-11].

Open reduction and internal fixation with a plate initially appear to be the treatment of choice because it addresses the drawbacks of conservative management. However, open wounds or severe closed soft-tissue injuries make an open procedure more difficult to perform, making external fixation the preferred option. HEF under the guidance of c-arm provides adequate reduction and fixation while limiting the complications associated with open reduction and internal fixation [12,13]. Some studies have reported that fractures managed with this technique have good clinical and radiological outcomes [13,14]. Although external fixation prevents future soft-tissue damage, there are still other possible concerns, such as pin-tract infections and poor patient compliance. Through the use of this method, soft-tissue injury is minimized, and higher union rates are demonstrated [15]. Anatomic reduction and stable fixation are preferred regardless of the treatment technique used [16]. In this study, two matched groups of patients with extra-articular proximal tibia metaphyseal fractures were treated at a single center with either hybrid external fixation or open reduction and internal fixation with bridge plating.

In our study, most were young adults, and the mean age of the HEF group was 40.48 ± 18.63 years and of the bridge plating group was 46.91 ± 17.69 years. Duwelius and Connolly [17] reported an average age of 48 years, and Porter [18] in his study reported an average age of 47 years, which were similar to the average age observations of our study.

There were 43 males and three females, indicating a male preponderance of the said fracture. In the HEF group, 100% were male, and in the plating group, 13.04% were female and 86.96% were male. In the present study, males were most affected, which was similar to the findings reported by Albuquerque et al. [19], Manidakis et al. [20], and Mehin et al. [21]. There was no significant difference in sex distribution between the two groups.

In this study, the bridge plating group consisted of 91.3% of patients who had injuries resulting from an RTA while 8.7% had been hit by an animal. The HEF group also had 91.3% of patients who were injured as a result of an RTA, but the remaining 4.35% each came following a fall of a heavy object on the leg and a fall from height, respectively. Ashwani et al. reported similar results with 92% of cases due to RTAs [22].

Mean surgery time was comparatively lesser in the HEF group which was 60.0 ± 9.53 minutes, and 71.09 ± 10.76 minutes in the bridge plating group which was significant as the p-value came out to be 0.001. In our study, the hospital stay time was more in the HEF group (5.48 days) patients compared to the bridge plating group (5.17 days) patients but this difference was not much significant between both groups. Similar results were discovered by the Canadian Orthopedic Trauma Society [23].

In our study, we found that in the HEF group, 21.74% of patients had a pin-tract infection which is more than in a study conducted by Babis et al. [24] (9%) and 4.35% had delayed union, while in the plating group, 8.7% suffered from screw back out, 4.35% had superficial wound infection, and 4.35% had wound gaping, which were lower than those reported in the study by Barei et al. [25] (8.4%) and Canadian Orthopedic Trauma Society (20%) [23]. There were different complications and different rates found in both the groups but, overall, pin-tract infection is a noticeable complication in the HEF group and superficial wound infection in the plating group. A comparative study done by us reflected that the bridge plating group had lesser complications (17.39%) as compared to the HEF group (26.09%).

In the randomized controlled study conducted by the Canadian Orthopedic Trauma Society, the average knee range of motion was 109° for plating and 120° for circular external fixators [23]. In our study, postoperative knee flexion in the bridge plating group turned out to be 117.30 and 103.70 in the HEF group after a six-month follow-up.

The mean KSS in the HEF group was 69.43 ± 8.11 and in the bridge plating group was 75.00 ± 8.22 with a p-value of 0.026. According to KSS, in the HEF group, 13.04% had excellent outcomes, 30.43% had good, 43.48% had fair, and 13.04% had a poor outcome. In the bridge plating group, 30.43% had an excellent outcome, 43.48% had a good, 21.74% had a fair, and 4.35% had a poor outcome. Similar results have been published by Chaix et al. as excellent to good scores were seen in 86% of the patients managed by surgical means [22] (Table 5).

**Table 5:** Final outcome distribution between two groups according to Knee Society Score.

		Group			
		Hybrid external fixator group		Bridge plating group	
		Count	%	Count	%
Final outcome using Knee Society Score	Excellent	3	13.04	7	30.43
	Good	7	30.43	10	43.48
	Fair	10	43.48	5	21.74
	Poor	3	13.04	1	4.35

This study did have certain restrictions. First, this study had a small sample size of 46 patients. Second, this was a single-center study, and third, the selection of fracture fixation modality depends on the type of fracture, degree of comminution, type of injury, i.e., open or closed, and quality of bone.

## Conclusions

Both bridge plating and HEF appear to be adequate fixation methods for proximal tibia metaphyseal fractures, and each of them has its pros and cons. In our comparative study, based on our results we saw that the HEF had some advantages like mean surgery time being less with minimal soft-tissue dissection, and PWB as well as FWB being commenced earlier in this group because of the earlier appearance of radiologically visible callus. However, on the other hand, bridge plating resulted in better postoperative knee ROM and gave a slightly better functional outcome compared to the HEF group according to the KSS system. We thus conclude that for treating proximal tibia metaphyseal fracture, bridge plating is a better treatment modality than the HEF but the type of fracture, degree of comminution, type of injury, i.e., open or closed, and quality of bone would also influence the clinical outcome.
